# Minimally invasive sleeve lobectomy for centrally located lung cancer: A real-world study with propensity-score matching

**DOI:** 10.3389/fonc.2023.1099514

**Published:** 2023-02-01

**Authors:** Tangbing Chen, Weigang Zhao, Chunyu Ji, Jizhuang Luo, Yiyang Wang, Yuan Liu, Walter Weder, Wentao Fang

**Affiliations:** ^1^ Department of Thoracic Surgery, Shanghai Chest Hospital, Shanghai Jiao Tong University School of Medicine, Shanghai, China; ^2^ Department of Thoracic Surgery, Shanghai Jiao Tong University School of Medicine Affiliated Sixth People’s Hospital, Shanghai, China; ^3^ Statistics Center, Shanghai Chest Hospital, Shanghai Jiao Tong University School of Medicine, Shanghai, China; ^4^ Division of Thoracic Surgery, University Hospital Zurich, Zurich, Switzerland

**Keywords:** sleeve lobectomy, minimally invasive surgery, thoracotomy, robot-assisted thoracoscopic surgery, video-assited thoracoscopic surgery

## Abstract

**Background:**

The safety, feasibility, and prognosis of sleeve lobectomy by minimally invasive surgery (MIS) remain to be validated. The purpose of this study was to investigate outcomes in real-world patients receiving minimally invasive sleeve lobectomy in a balanced large cohort.

**Methods:**

Between January 2013 and December 2018, 578 consecutive patients undergoing sleeve resection at a high-volume center were retrospectively analyzed. Surgical and oncologic outcomes were compared between MIS and thoracotomy patients after propensity-score matching (PSM).

**Results:**

MIS sleeve lobectomy was increasingly used as a time-trend in real-world. Before PSM, the MIS group had smaller tumor size, more T2-stage cases, and more right upper lobe sleeve lobectomies compared to the Open group. After 1:4 PSM by patient demographics and tumoral characteristics, 100 cases of MIS and 338 cases of Open sleeve lobectomy were further analyzed. Although median operation time was longer in the MIS group than in the Open group (170.5 minutes vs.149.5 minutes, P < 0.001), patients in MIS group had significantly less estimated intraoperative blood loss (100 ml vs. 200 ml, P = 0.003), shorter drainage duration (5 days vs. 6 days, P = 0.027) and less amount of drainage (1280 ml vs. 1640 ml, P < 0.001) after surgery. Complete resection rate, combined angioplasty, number of dissected lymph nodes, post-operative length of stay, postoperative morbidity and mortality rate, and application of adjuvant therapy were similar between the two matched groups. Conversion to open thoracotomy was necessary in 13.6% patients, but with similar perioperative outcomes compared to Open cases except for longer operation time. More lower lobe sleeve lobectomies were accomplished *via* robot-assisted thoracoscopic surgery than *via* video-assisted thoracoscopic surgery (40.0% vs. 12.0%, P = 0.017) in MIS patients. Five-year overall survivals (MIS vs. Open: 72.7% vs. 64.4%, P = 0.156) and five-year progression-free survivals (MIS vs. Open: 49.2% vs. 50.5%, P = 0.605) were similar between the two matched groups.

**Conclusions:**

MIS sleeve lobectomy is associated with similar or even better perioperative results and oncologic outcomes to open thoracotomy. Conversion to thoracotomy does not compromise perioperative outcomes. Robot surgery may be preferable for more complex sleeve resections.

## Introduction

Lung cancer is currently one of the leading causes of cancer death in the world (1). Non-small cell lung cancers (NSCLCs) can be clinically divided into centrally located and peripheral ones according to their position in the lung. In 1933, Graham performed the first successful pneumonectomy (2) for a centrally located lung cancer. However, pneumonectomy is associated with high mortality and morbidity. A selected group of centrally located tumors can be completely resected by using bronchoplastic techniques with anastomosis of one lobar bronchus to the other to preserve lung parenchyma. These so-called sleeve resections were reported for the first time by Clement Price Thomas in 1956 (3). Compared to pneumonectomy, sleeve lobectomy has been shown to be associated with less morbidity and mortality but similar or even better long-term survival if the tumor could be completely removed (4–10). It has thus become the preferred surgical procedure for centrally located NSCLC, whenever technically feasible and when complete resection can be achieved (11).

Minimally invasive surgery (MIS), including video-assisted thoracoscopic surgery (VATS) and robot-assisted thoracoscopic surgery (RATS), is the preferred approach in the current guidelines for the surgical management of early-stage NSCLC (12). Its advantage over open thoracotomy includes less pain, decreased postoperative complications, less impaired pulmonary function, and better quality of life and compliance to adjuvant therapies after surgery (13–16). And similar oncologic outcomes in lymph node dissection and long-term survival have been demonstrated in MIS and open surgery (17, 18). But most sleeve lung resections are still accomplished *via* conventional open thoracotomy, as they are technically more demanding and are often applied in locally advanced tumors. Although Santambrogio et al. (19) reported the first successful case of VATS sleeve lobectomy in 2002, up till now, there have been only a few single-institutional reports with small numbers of cases showing its feasibility technically (20–30). Although the conversion rates reported in these series were generally higher than in standard lobectomy, converted cases were either excluded or their outcomes not studied in the previous reports. Most reported cases were accomplished *via* VATS, with very few RATS cases included (27, 29). Since most of the MIS sleeve lobectomies were done in recent years, follow-up time of MIS patients was unanimously short in these series. Therefore, the safety and efficacy of MIS in sleeve lobectomy for NSCLC remains largely unknown.

Our study thus aimed to find out the results of MIS for sleeve lobectomy in a real-world setting, with special attention paid to its potential benefits and surgical outcomes in conversion cases, and to the unique advantages of RATS. 

## Materials and methods

Patients with centrally located primary NSCLC receiving bronchial sleeve resection with or without pulmonary artery angioplasty at the Shanghai Chest Hospital between January 2013 and December 2018 were retrospectively identified from the institutional database. The study was approved by the Institutional Review Board of Shanghai Chest Hospital (No. KS(Y) 21268). Informed consent was waived as only de-characterized data were used for the study.

Designed as a real-world study, all consecutive patients receiving sleeve lobectomy for potentially resectable primary NSCLC *via* either MIS or Open thoracotomy were included. Exclusion criteria were concomitant carina resection or reconstruction of great vessels such as superior vena cava, patients with metastatic disease, small cell lung cancer, or benign diseases. All patients were confirmed of having central lung cancer by bronchoscopy. Pretreatment evaluation included chest computed tomography (CT) scan, brain magnetic resonance imaging, neck, and abdominal ultrasonography, bone scintigraphy, or positron emission tomography. Tumor stage was re-classified according to the 8th Edition TNM Classification of Malignant Tumors (31).

Patients were divided into two groups according to the planned surgery: the MIS group and the Open group. The approach was chosen according to the surgeons’ decision. Those converted to open thoracotomy during the operation were included in the MIS group, using an intention-to-treat (ITT) analysis.

General anesthesia and double-lumen tube intubation were used in all patients. Open thoracotomy was performed using a postero-lateral incision at the fourth or fifth intercostal space. MIS was accomplished *via* one, two, three, or four-port VATS or RATS according to surgeons’ preference. Frozen section was performed intraoperatively in all patients to assess the resected bronchial margins. In patients with poor pulmonary function or severe comorbidity, pneumonectomy was usually avoided even if the bronchial margin was positive, and postoperative radiotherapy would be recommended to these patients. Most of the surgeons in our institution chose to do the running sutures using non-absorbable thread in MIS and Open sleeve lobectomy. But a few surgeons preferred absorbable thread to do interrupted sutures in open cases. Angioplasty would be added if the pulmonary artery trunk was also invaded by the tumor. In open sleeve cases, some surgeons preferred covering the anastomosis with muscle or pericardium flap or thymus. However, we do not routinely cover the anastomosis with any tissues in the MIS sleeve cases. We routinely did bronchoscopy right after finishing the bronchial anastomosis to control the anastomosis.

After surgery, adjuvant chemotherapy was recommended to patients with histologically proven stage II or III diseases who did not receive neoadjuvant therapy before surgery. Adjuvant radiation would also be suggested to patients with positive resection margin or pathological N2 disease. Patients were followed every three months after treatment in the first two years and 6-12 months afterwards. These routinely included serum tumor markers, chest CT scan, neck, and abdominal ultrasonography. Brain magnetic resonance imaging, bone scintigraphy, or positron emission tomography was conducted when disease progression was suspected.

Patients’ demographics, tumoral characteristics, and treatment outcomes were compared between the two groups. Overall survival (OS) was defined as the duration from the date of operation to death of any cause or the date of last follow-up. Progression-free survival (PFS) was defined as the duration from the date of operation to the date of progress or death of any cause or the date of last follow-up.

Continuous variables were expressed as mean ± standard deviations (SD) if normally distributed, otherwise were exhibited as median with interquartile range (IQR). Student t-test or Mann-Whitney test was used for comparison. Comparison of categorical variables was performed by Chi-Squared test or Fisher’s exact test when appropriate. Survival curves were plotted using the Kaplan-Meier method. Log-rank test was used to compare survivals between different groups. As the baseline characteristics in the MIS and Open cases were not balanced, a propensity-score matched (PSM) analysis was performed with R version 4.2.0. A 1:4 matching was performed by potential confounding factors including sex, age, body mass index (BMI), forced expiratory volume in one second (FEV1), percentage of diffusing capacity of the lung for carbon monoxide (DLCO%), comorbidity, smoking history, tumor size, tumor location, clinical T and N stage, histological classification, and neoadjuvant chemotherapy. Patients having MIS were ordered and sequentially matched to the nearest unmatched patients having thoracotomy. Surgical and postoperative outcomes were then compared between the matched groups. Univariable analysis was performed using the Cox univariate model to assess the impact of potential risk factors on survival and disease-progression. Multivariable analysis was performed with a Cox proportional model, using the enter method. The variables would be included into the multivariable analysis if their P-values were less than 0.05 in univariable analysis. Statistical significance was defined as P < 0.05 throughout the study.

## Results

**Figure 1:**
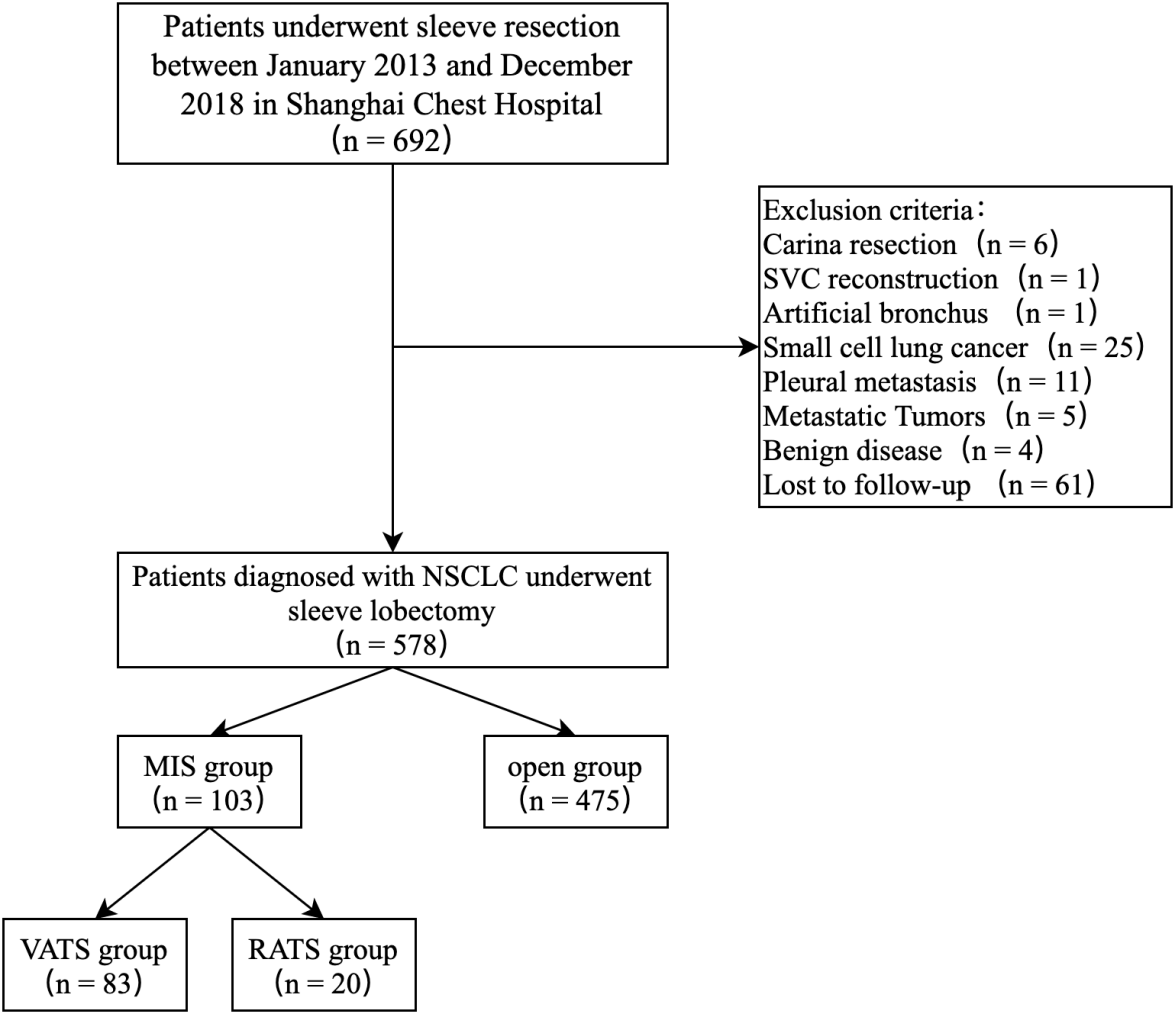
Study population flow diagram — patients who underwent sleeve lobectomy between 2013 and 2018.

**Figure 2:**
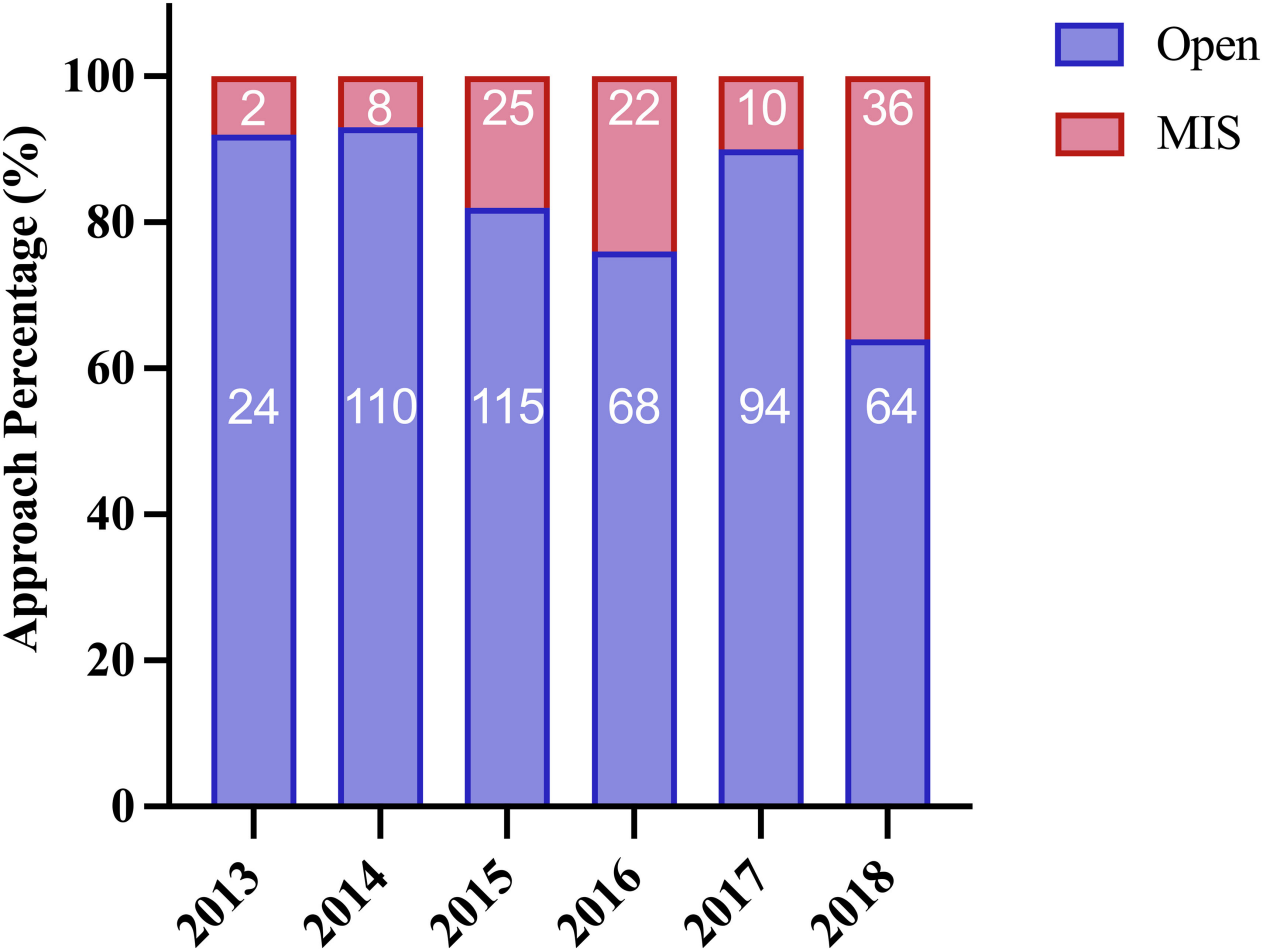
Annual numbers and percentage of patients underwent MIS and open sleeve lobectomy.

Between January 2013 and December 2018, 692 consecutive patients underwent sleeve lobectomy at the Shanghai Chest Hospital. Based on the exclusion criteria, 114 cases were excluded, leaving 578 patients for analysis, 103 (17.8%) in the MIS group and 475 (82.2%) in the Open group. The MIS group included 20 RATS cases and 83 VATS cases (**Figure 1**). There was an obvious trend toward increasing use of MIS in sleeve lobectomy patients during the study period (7.7% in 2013 to 36% in 2018), as shown in **Figure 2**. Conversion to thoracotomy was found necessary in 14 patients (13.6%) due to difficult tumor or hilar lymph nodes, dense adhesion, or unexpected intraoperative bleeding. Details of patient demographics and oncologic characteristics are listed in **Table 1**. Demographic characteristics were similar between the MIS and the Open groups before PSM. There was no difference in patient age, sex, comorbidity or functional status between the MIS and the Open group. The proportion of patients receiving neoadjuvant therapy (10.7% vs. 16.0%, P = 0.224) were also similar. However, right upper lobe sleeve lobectomy accounted for only 47.2% of the cases in the Open group, while it was 59.2% in the MIS group (P = 0.026). There was only one (0.9%) case of sleeve bilobectomy in the MIS group but 17(3.6%) in the Open group. The MIS group also had more patients with smaller lesions and thus more cT2 tumors (94.2% vs. 85.9%, P = 0.022). The mean diameter of tumor was 32 mm in the MIS group and 37 mm in the Open group (P = 0.002).

**Table 1:** Demographic and pathologic characteristics before and after propensity-score matching.

Characteristics	Unmatched cohort			Matched cohort		
	Open (n=475)	MIS (n=103)	*P*	Open (n=338)	MIS (n=100)	*P*
Sex, n (%)			0.601			0.907
Male	439 (92.4)	93 (90.3)		311 (92.0)	91 (91.0)	
Female	36 (7.6)	10 (9.7)		27 (8.0)	9 (9.0)	
Age (year), mean ± SD	61.2 ± 8.8	60.2 ± 8.5	0.290	60.6 ± 8.5	60.4 ± 8.4	0.861
BMI (kg/m^2^), mean ± SD	23.3 ± 3.0	23.0 ± 3.1	0.333	23.2 ± 2.8	23.0 ± 3.0	0.691
CCI, n (%)			0.514			0.703
0	356 (74.9)	80 (77.7)		255 (75.4)	77 (77.0)	
1	86 (18.1)	19 (18.4)		62 (18.3)	19 (19.0)	
≥2	33 (6.9)	4 (3.9)		21 (6.2)	4 (4.0)	
Smoking history, n (%)			0.754			0.888
Never	252 (53.1)	57 (55.3)		181 (53.6)	55 (55.0)	
Ever	223 (46.9)	46 (44.7)		157 (46.4)	45 (45.0)	
FEV1%, mean ± SD	79.4 ± 14.9	81.9 ± 14.6	0.127	80.2 ± 15.2	81.9 ± 14.8	0.318
DLCO%, mean ± SD	86.7 ± 18.6	87.1 ± 16.0	0.859	86.0 ± 17.4	87.3 ± 16.2	0.503
Histology, n (%)			0.385			0.486
Squamous	372 (78.3)	76 (73.8)		270 (79.9)	76 (76.0)	
Non-suamous	103 (21.7)	27 (26.2)		68 (20.1)	24 (24.0)	
Location, n (%)			0.026			0.118
RUL	224 (47.2)	61 (59.2)		166 (49.1)	58 (58.0)	
others	251 (52.8)	42 (40.8)		172 (50.9)	42 (42.0)	
Size (mm), mean ± SD	37.3 ± 16.2	32.0 ± 14.3	0.002	33.9 ± 13.5	32.7 ± 13.9	0.424
Clinical T stage, n (%)			0.022			0.508
cT2	408 (85.9)	97 (94.2)		311 (92.0)	94 (94.0)	
cT3 + cT4	67 (14.1)	6 (5.8)		27 (8.0)	6 (6.0)	
Clinical N stage, n (%)			0.251			0.612
cN0	214 (45.1)	55 (53.4)		162 (47.9)	52 (52.0)	
cN1	217 (45.7)	38 (36.9)		147 (43.5)	38 (38.0)	
cN2	44 (9.3)	10 (9.7)		29 (8.6)	10 (10.0)	
Neoadjuvant therapy, n (%)	76 (16.0)	11 (10.7)	0.224	42 (12.4)	11 (11.0)	0.834

MIS, minimally invasive surgery; SD, standard deviation; BMI, body mass index; FEV1, forced expiratory volume in 1 second; DLCO, carbon monoxide diffusing capacity; CCI, Charlson Comorbidity Index; RUL, right upper lobe; RML, right middle lobe; RLL, right lower lobe; LUL, left upper lobe; LLL, left lower lobe.

In the unmatched cohort, median operation time was 171 minutes in the MIS group and 151 minutes in the Open group (P < 0.001). However, rate of angioplasty (7.8% vs. 8.4%, P = 0.983), R0 resection (84.5% vs. 84.6%, P = 0.966) and median number of harvested lymph nodes (15 vs. 15, P = 0.445) were similar between the two groups. The MIS group had shorter drainage duration (5 days vs. 6 days, P = 0.004), less drainage amount (1270 ml vs. 1670 ml, P < 0.001) and shorter length of postoperative hospitalization (7 days vs. 8 days, P = 0.007) compared to the Open group. In term of postoperative complications, there was only one patient diagnosed with bronchopleural fistula (BPF) after surgery in the MIS group who recovered after conservative treatment. Regarding the late complications, there was 1 patient in the MIS group and 2 in the Open group experiencing bronchial stenosis after surgery. These patients received balloon dilation *via* bronchoscopy. We did not see any patients with late dehiscence postoperatively. Five patients in the Open group died due to bronchopleural fistula or pulmonary infection, and one in the MIS group died due to empyema and sepsis during postoperative hospitalization. The in-hospital mortality was not significantly different (MIS vs. Open, 1.0% vs. 1.1%, P = 0.940).

After PSM, 100 MIS and 338 Open patients were retained for further analysis. There was no longer any significant difference in patient demographics or tumor characteristics between the two matched groups (**Table 1**). Potential confounders like tumor size, tumor location, tumor stage, and proportion of patients receiving neoadjuvant therapy before surgery were well-balanced after PSM. In the matched cohort, median operation time in the MIS group was still longer than in the Open group (170.5 minutes [IQR, 134–224.5] vs.149.5 minutes [IQR, 128–179], P < 0.001). However, the estimated intraoperative blood loss was significantly less in the MIS group (100 ml [IQR, 100–200] vs. 200 ml [IQR, 100–200], P = 0.003). There was no difference in complete resection rate, number of total or mediastinal lymph nodes dissected between the two groups. After surgery, chest drainage duration (5 days [IQR, 4–7] vs. 6 days [IQR, 5–7], P = 0.027) was significantly shorter, and total amount of drainage (1280 ml [IQR, 957.5–1695] vs. 1640 ml [IQR, 1200–2307.5], P < 0.001) was significantly less in the MIS group than in the Open group. Postoperative ICU stay and length of stay in hospital was similar (**Table 2**) between two groups. The overall postoperative complication rate was 18.0% in the MIS group and 20.7% in the Open group, which was also similar (**Table 2**). The BPF rate was 0% in the MIS group and 1.5% in the Open group in the matched cohort.

**Table 2:** Perioperative outcomes before and after propensity-score matching.

Characteristics	Unmatched cohort			Matched cohort		
	Open (n=475)	MIS (n=103)	*P*	Open (n=338)	MIS (n=100)	*P*
Operating time (minute), (median (IQR))	151 (127, 179)	171 (134, 227)	<0.001	149.5 (128, 179)	170.5 (134, 224.5)	<0.001
Intraoperative blood loss (ml), (median (IQR))	200 (100, 200)	200 (100, 200)	0.001	200 (100, 200)	100 (100, 200)	0.003
Angioplasty, n (%)	40 (8.4)	8 (7.8)	0.983	25 (7.4)	8 (8.0)	0.841
R0, n (%)	402 (84.6)	87 (84.5)	0.966	282 (83.4)	84 (84.0)	0.893
LN numbers (median (IQR))	15 (11, 20)	15 (11, 20)	0.445	15.5 (11, 20.75)	15 (11, 20)	0.424
MLN numbers (median (IQR))	9 (6, 12)	8 (5, 12)	0.483	9 (6, 12)	8 (5, 12)	0.443
Pathological T stage, n (%)			0.010			0.179
pT0	0 (0.0)	1 (1.0)		0 (0.0)	1 (1.0)	
pT2	394 (82.9)	95 (92.2)		301 (89.1)	92 (92.0)	
pT3	63 (13.3)	6 (5.8)		28 (8.3)	6 (6.0)	
pT4	18 (3.8)	1 (1.0)		9 (2.7)	1 (1.0)	
Pathological N stage, n (%)			0.211			0.508
pN0	209 (44.0)	55 (53.4)		157 (46.4)	52 (52.0)	0.503
pN1	148 (31.2)	28 (27.2)		96 (28.4)	28 (28.0)	0.486
pN2	118 (24.8)	20 (19.4)		85 (25.1)	20 (20.0)	
ICU stay(day), (median (IQR))	1 (0, 3)	1 (0, 3)	0.237	1 (0, 3)	1 (0, 3)	0.338
Drainage duration (day), (median (IQR))	6 (5, 8)	5 (4, 7)	0.004	6 (5, 7)	5 (4, 7)	0.027
Drainage amount (ml), (median (IQR))	1670 (1235, 2350)	1270 (910, 1680)	<0.001	1640 (1200, 2307.5)	1280 (957.5, 1695)	<0.001
LOS (day), (median (IQR))	8 (7, 10)	7 (6, 9)	0.007	8 (7, 10)	7 (6, 9)	0.053
Complication in hospital, n (%)	97 (20.4)	18 (17.5)	0.587	70 (20.7)	18 (18.0)	0.651
Prolonged air leak	27 (5.7)	4 (3.9%)		19 (5.6)	4 (4.0)	
Arrhythmia	17 (3.6)	5 (4.9%)		13 (3.8)	5 (5.0)	
Pulmonary infection	15 (3.2)	2 (1.9%)		9 (2.7)	1 (1.0)	
Atelectasis	15 (3.2)	6 (5.8%)		12 (3.6)	6 (6.0)	
Bronchopleural fistula	9 (1.9)	1 (1.0%)		5 (1.5)	0 (0.0)	
Empyema	4 (0.8)	1 (1.0%)		4 (1.2)	1 (1.0)	
Respiratory failure	3 (0.6)	1 (1.0%)		2 (0.6)	0 (0.0)	
Hemothorax	3 (0.6)	0 (0.0%)		3 (0.9)	0 (0.0)	
Chylothorax	2 (0.4)	0 (0.0%)		1 (0.3)	0 (0.0)	
Mortality in hospital, n (%)	5 (1.1)	1 (1.0)	0.940	5 (1.5)	1 (1.0)	0.707
Adjuvant chemotherapy, n (%)	220 (46.3)	51 (49.5)	0.631	153 (45.3)	51 (51.0)	0.372
Adjuvant radiotherapy, n (%)	67 (14.1)	17 (16.5)	0.637	51 (15.1)	17 (17.0)	0.759

MIS, minimally invasive surgery; LN, lymph node; MLN, mediastinal lymph node; ICU, intensive care unit; LOS, length of stay.

Perioperative outcomes were also compared between the conversion patients and the Open group. The conversion cases had longer operation time than the Open group (190.5 minutes [IQR, 153.25–306.25] vs. 151 minutes [IQR, 127–179], P = 0.004). However, intraoperative blood loss, number of total and mediastinal lymph nodes dissected, postoperative ICU stay, postoperative drainage duration or amount, postoperative length of hospitalization, or postoperative complication rates were similar between the two groups. No conversion cases died during hospitalization (**Table 3**).

**Table 3:** Perioperative outcomes between conversion and open groups in unmatched cohort.

Characteristics	Conversion (n=14)	Open (n=475)	*P*
Operating time (minute), (median (IQR))	190.5 (153.25, 306.25)	151 (127, 179)	0.004
Intraoperative bleeding (ml), (median (IQR))	200 (200, 300)	200 (100, 200)	0.152
Angioplasty, n (%)	3 (21.4)	40 (8.4)	0.224
R0, n (%)	13 (92.9)	402 (84.6)	0.642
LN numbers (median (IQR))	15.5 (13, 20)	15 (11, 20)	0.600
MLN numbers (median (IQR))	8 (7, 11.5)	9 (6, 12)	0.849
Pathological T stage, n (%)			0.758
pT2	12 (85.7)	394 (82.9)	
pT3	2 (14.3)	63 (13.3)	
pT4	0 (0.0)	18 (3.8)	
Pathological N stage, n (%)			0.724
pN0	7 (50.0)	209 (44.0)	
pN1	3 (21.4)	148 (31.2)	
pN2	4 (28.6)	118 (24.8)	
ICU stay(day), (median (IQR))	1.5 (0.25, 3)	1 (0, 3)	0.738
Length of drainage (day), (median (IQR))	6 (4.25, 7.50)	6 (5, 8)	0.341
Drainage amount (ml), (median (IQR))	1435 (855, 1972.5)	1670 (1235, 2350)	0.122
LOS (day), (median (IQR))	7 (6, 8.75)	8 (7, 10)	0.065
Complication in hospital, n (%)	1 (7.1)	97 (20.4)	0.376
Mortality in hospital, n (%)	0 (0.0)	5 (1.1)	0.589
Adjuvant chemotherapy, n (%)	7 (50.0)	220 (46.3)	0.785
Adjuvant radiotherapy, n (%)	4 (28.6)	67 (14.1)	0.259

LN, lymph node; MLN, mediastinal lymph node; ICU, intensive care unit; LOS, length of stay.

Patient characteristics and perioperative outcomes were further compared between VATS and RATS cases (**Table 4**). Operation time, intraoperative blood loss, lymph node dissection, postoperative drainage, postoperative length of hospitalization and overall postoperative complication rates were similar between the two groups. However, significantly more lower lobe sleeve lobectomies were accomplished *via* RATS than *via* VATS (40.0% vs. 12.0%, P = 0.017).

**Table 4:** Perioperative outcomes between robot-assisted thoracic surgery and video-assisted thoracic surgery groups in unmatched cohort.

Characteristics	RATS (n=20)	VATS (n=83)	*P*
Operating time (minute), (median (IQR))	153 (118, 199.25)	172 (136.5, 233.5)	0.128
Intraoperative bleeding (ml), (median (IQR))	150 (100, 200)	200 (100, 200)	0.252
Angioplasty, n (%)	2 (10.0)	6 (7.2)	0.687
R0, n (%)	20 (100.0)	67 (80.7)	0.073
LN numbers (median (IQR))	15 (13, 18)	16 (10, 20)	0.655
MLN numbers (median (IQR))	9 (7.75, 11.25)	8 (5, 13)	0.496
Location, n (%)			0.017
RUL	7 (35.0)	54 (65.1)	
RML	0 (0.0)	1 (1.2)	
RLL or RML+RLL	1 (5.0)	0 (0.0)	
LUL	5 (25.0)	18 (21.7)	
LLL	7 (35.0)	10 (12.0)	
Pathological T stage, n (%)			0.867
pT2	19 (95.0)	76 (92.7)	
pT3	1 (5.0)	5 (6.1)	
pT4	0 (0.0)	1 (1.2)	
Pathological N stage, n (%)			0.311
pN0	8 (40.0)	47 (56.6)	
pN1	6 (30.0)	22 (26.5)	
pN2	6 (30.0)	14 (16.9)	
ICU stay(day), (median (IQR))	0.5 (0, 2.25)	1 (0, 3)	0.344
Length of drainage (day), (median (IQR))	5 (4, 6)	5 (4, 7)	0.667
Drainage amount (ml), (median (IQR))	1140 (695, 1492.5)	1300 (955, 1680)	0.353
LOS (day), (median (IQR))	8 (6, 9)	7 (6, 9)	0.671
Complication in hospital, n (%)	3 (15.0)	15 (18.1)	0.742
Mortality in hospital, n (%)	1 (5.0)	0 (0.0)	0.437
Adjuvant chemotherapy, n (%)	6 (30.0)	45 (54.2)	0.091
Adjuvant radiotherapy, n (%)	2 (10.0)	15 (8.1)	0.591

RATS, robot-assisted thoracoscopic surgery; VATS, video-assisted thoracoscopic surgery; RUL, right upper lobe; RML, right middle lobe; RLL, right lower lobe; LUL, left upper lobe; LLL, left lower lobe; LN, lymph node; MLN, mediastinal lymph node; ICU, intensive care unit; LOS, length of stay.

**Figure 3:**
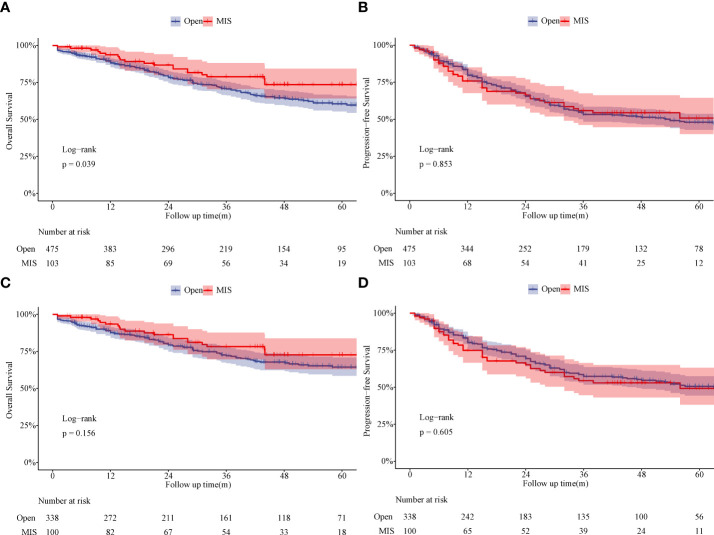
Comparison of overall survival and progression-free survival between the Open group and MIS group. **(A)** Comparison of overall survival between the Open group and MIS group (unmatched). **(B)** Comparison of progression-free survival between the Open group and MIS group (unmatched). **(C)** Comparison of overall survival between the Open group and MIS group (matched). **(D)** Comparison of progression-free survival between the Open group and MIS group (matched).

**Figure 4:**
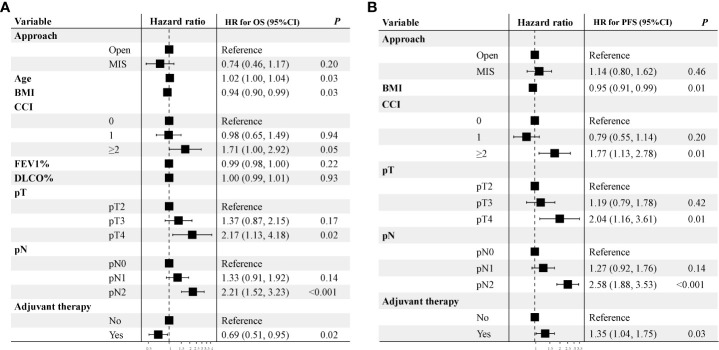
Multivariable analysis of overall survival and progression-free survival of unmatched cohort. A hazard ratio more than 1 implies a higher risk of overall survival and progression-free survival after sleeve lobectomy.

The median follow-up time was 31 months in the Open group and 42 months in the MIS group before PSM. Five-year OS rate in the MIS group was significantly better than in the Open group (73.5% vs. 60.6%, P = 0.039) before PSM (**Figure 3A**). Five-year PFS rate was 47.9% in the MIS group and 50.7% in the Open group without significant difference before PSM (**Figure 3B**). As showed in **Figure 3C**, five-year OS remained better in the MIS group compared with the Open group after PSM, although without statistical significance (72.7% vs. 64.4%, P = 0.156). Five-year PFS was similar after PSM, 49.2% in the MIS group and 50.5% in the Open group (**Figure 3D**). To determine whether surgical approach would have any impact on OS and PFS, univariable and multivariable analyses were performed in the entire cohort (**Supplemental Table S1**). The multivariable results showed that surgical approach was not associated with OS or PFS in sleeve lobectomy patients (**Figure 4**). Interestingly, we also found that there was no significant difference in five-year OS rates (60.7% vs. 63.1%, P = 0.763) or PFS rates (39.1% vs. 49.9%, P = 0.205) between margin positive group and margin negative group. When we delved into the database, we found that 37(41.6%) patients received postoperative radiotherapy (PORT) in margin positive group for local control, but only 47(9.6%) patients in the margin negative group received PORT.

## Discussion

In this real-world study, only 17.8% of the sleeve resections for NSCLC were performed by MIS. MIS including both VATS and RATS were increasingly used during the study period, although it was more often used for smaller tumors and relatively simpler right upper lobe sleeve resections. Our study showed that in a matched cohort, intraoperative blood loss and postoperative drainage after MIS sleeve lobectomy was significantly less than after open surgery, although operation time for sleeve lobectomy by MIS was about 20 minutes longer than by open surgery. And overall mortality and morbidity were comparable between the two groups. The conversion cases had similar postoperative outcomes compared to the Open cases. What is more, there was no significant difference between the MIS group and the Open group in OS or PFS. And surgical approach was not associated with long-term outcomes in multivariable analysis for survivals.

In the real-world, the application of MIS for sleeve lobectomy was still much less often used than open thoracotomy even at a high-volume thoracic surgery center. According to the annual report of the Shanghai Chest Hospital, the overall MIS rate for lung surgery was over 95% (32), but the MIS rate for sleeve lobectomy was only 17.8% in our study during the same time period. As a technically demanding procedure, surgeons tended to perform MIS sleeve lobectomy for smaller tumors in earlier stages or less complex right upper lobe sleeve lobectomy, as was shown in our study. However, pulmonary function and comorbidity had no influence on patient selection. Unlike most previous reports, patients with neoadjuvant therapies before surgery or requiring angioplasty were also included in our study. Even with such more difficult MIS cases (10.7% after neoadjuvant therapy and 7.8% of angioplasty), perioperative results and long-term survivals were not compromised or even better after MIS sleeve lobectomy than after open surgery in the real world.

To reduce potential selection bias, we performed PSM and ITT analysis to validate our findings. In the matched cohort, although operation time by MIS was around 20 minutes longer than that by open surgery, it did not bring any additional complication compared to open surgery. This was further supported by the very low rate of anastomotic complications, especially BPF, which occurred similarly between the MIS group and the Open group (1.0% vs. 1.9%). On the other hand, intraoperative blood loss and postoperative amount of drainage in the MIS group were significantly less than in the Open group, indicating that MIS sleeve lobectomy could render uncompromised recovery and carries with it certain benefits in selected patients with centrally located NSCLC. Recently a database study showed that the VATS approach was associated with shorter length of stay and decreased morbidity in sleeve lobectomy cases (33), which was consistent with our findings.

In this study, there were fourteen cases intended to receive MIS but were converted to open surgery. The conversion rate was 13.6%, which was similar to the 4.5%–21.1% conversion rates in the other published MIS sleeve lobectomy studies (28–30, 33). Unfortunately, none of those studies reported the surgical outcomes in converted cases. Our results showed that although operation time was longer in the conversion cases than open surgery, intraoperative blood loss, postoperative drainage, length of hospital stays, and postoperative complication rates were similar between the two groups. No conversion patient died after surgery during hospital stay. Our results suggested that conversion to thoracotomy during the operation did not bring additional risks to the patients. It is thus safe and feasible to start sleeve lobectomy minimally invasively in well selected patients.

Robotic surgery has gradually become an integrated part of MIS. However, whether RATS had any advantages in sleeve lobectomy remains to be explored. Among all sleeve lobectomies, right upper lobe is the most straight forward. The lower lobe sleeve lobectomies are comparatively more complex because of the anastomosis angles and greater size discrepancy between the proximal and distal bronchi. In addition to significantly more right upper sleeve lobectomies in the MIS group than in the Open group (59.2% vs. 47.2%), percentage of right upper sleeve lobectomy was the highest in VATS cases (65.1%) but was the lowest (35.0%) in RATS cases. Meanwhile 40.0% lower lobe sleeve lobectomies were done *via* RATS, but only 12.0% of them were done *via* VATS. This was in consistency with the findings in Qiu’s study in which lower lobe sleeve lobectomies were most often accomplished *via* RATS than *via* VATS or open thoracotomy (26.5% vs. 21.9% vs. 16.7%) (29). There are two potential explanations for this. First, RATS is more flexible and feasible than VATS. The three-dimensional and magnified vision and the dexterous robotic arms are helpful in more demanding cases. Second, surgeons favoring robotic surgery may be more experienced in MIS and in handling anastomotic difficulties. According to our previous study, short-term and mid-term outcomes after RATS sleeve lobectomy were comparable to open surgery (27). Therefore, RATS may be an important alternative in complex MIS surgery such as lower lobe sleeve lobectomy.

Previous studies suggested that oncological outcomes after MIS might be similar to open surgery in patients with NSCLC needing sleeve lobectomy. But one of the major limitations in most such studies was the relatively short follow-up time in MIS patients, being 24–36.8 months in previous published reports (28–30). This was mostly because MIS sleeve lobectomies were often accomplished more recently, with open cases in earlier years as historical controls. The median follow-up time of the MIS group reached 42 months in our study. And it is by far the longest follow-up time in MIS sleeve lobectomy patients, with a control Open group during the same time period. OS turned out to be significantly better in the MIS group than in the Open group (73.5% vs. 60.6%, P = 0.039), probably because of more smaller tumors in MIS patients. Although OS in the MIS group was still better than in the Open group after PSM, PFS was similar between the two groups both before (47.9% vs. 50.7%, P = 0.853) and after PSM (49.2% vs. 50.5%, P = 0.605). Together with similar R0 resection rates and numbers of lymph node dissected, our results indicated that oncological outcomes after minimally invasive sleeve lobectomy were at least non-inferior to those after open thoracotomy. There have been studies showing that MIS approach could reduce level of cytokine responses and lead to better immune function than open surgery (34, 35). Hopefully with increasing experience in MIS, sleeve lobectomy patients may have benefit in both perioperative recovery as well as prolonged survival in the future.

There were certain limitations in our study. First, our study was retrospective in nature. Unknown confounding factors like surgeons’ preference and expertise would still influence the results even though PSM was used to diminish potential impact from patient conditions and tumor characteristics. However, this study included all consecutive patients receiving sleeve lobectomy for potentially resectable primary NSCLC, using an ITT analysis. Our study results clearly revealed the surgical and oncological outcomes of MIS in the real world. Second, all patients included in this study were treated at a single institution, which has a very high surgical volume and has more experience in MIS for lung cancers. It would thus be interesting to use external data to validate our findings on the efficacy of MIS sleeve lobectomy. Third, the detailed information on conversion to pneumonectomy could not be accurately retrieved due to the retrospective nature of the study. However, we found that the positive margin did not compromise the long-term survival of sleeve lobectomy patients, probably because of the role of postoperative radiation for local control.

## Conclusions

In conclusion, MIS sleeve lobectomy is still a technically demanding procedure currently. Nonetheless, it is safe and feasible in experienced hands, with similar or even better surgical and oncologic outcomes compared to open surgery in well-selected patients. And RATS may be preferable for more difficult sleeve cases. Conversion to thoracotomy does not compromise perioperative recovery of the patients. Therefore, it does little harm to try sleeve lobectomy minimally invasively first.

## Data availability statement

The original contributions presented in the study are included in the article/**Supplementary Material**. Further inquiries can be directed to the corresponding author.

## Ethics statement

The studies involving human participants were reviewed and approved by the Institutional Review Board of Shanghai Chest Hospital. Written informed consent for participation was not required for this study in accordance with the national legislation and the institutional requirements.

## Author contributions

(I) Conception and design: TC, WZ, WF. (II) Administrative support: WF. (III) Provision of study materials or patients: TC, CJ. (IV) Collection and assembly of data: WZ, TC. (V) Data analysis and interpretation: All authors. (VI) Manuscript writing: All authors. (VII) Final approval of manuscript: All authors. All authors contributed to the article and approved the submitted version.
