# Supplementary material for: Minimally invasive sleeve lobectomy for centrally located lung cancer: A real-world study with propensity-score matching
Source: Front Oncol. 2023 Feb 1;13:1099514. doi: 10.3389/fonc.2023.1099514 (PMC9929062; doi:10.3389/fonc.2023.1099514)
Supplement: Supplementary file 1 [file Table_1.docx]

Supplemental Table S1. Univariable and multivariable analysis of overall survival and progression-free survival in unmatched cohort

| **Variables** | **OS** | | | | **PFS** | | | |
| --- | --- | --- | --- | --- | --- | --- | --- | --- |
|  | **Univariable analysis** | | **Multivariable analysis** | | **Univariable analysis** | | **Multivariable analysis** | |
|  | **HR (95%CI)** | ***P*** | **HR (95%CI)** | ***P*** | **HR (95%CI)** | ***P*** | **HR (95%CI)** | ***P*** |
| Approach (open) | 0.63 (  0.4 – 0.99  ) | 0.047 | 0.74 (0.46 –1.17) | 0.200 | 0.95 (0.68 – 1.33) | 0.763 | 1.14 (0.80 – 1.62) | 0.460 |
| Sex (male) | 0.98 (  0.57 – 1.7  ) | 0.956 |  |  | 0.78 (0.47 – 1.3) | 0.344 |  |  |
| Age | 1.02 (  1.01 – 1.04  ) | 0.011 | 1.02 (1 – 1.04) | 0.029 | 1.01 (1 – 1.03) | 0.066 |  |  |
| BMI | 0.94 (  0.89 – 0.99  ) | 0.014 | 0.94 (0.9 – 0.99) | 0.029 | 0.95 (0.91 – 0.99) | 0.022 | 0.95 (0.91 – 0.99) | 0.010 |
| CCI |  |  |  |  |  |  |  |  |
| 1 vs. 0 | 0.85 (  0.57 – 1.27  ) | 0.424 | 0.98 (0.65 – 1.49) | 0.939 | 0.68 (0.48 – 0.97) | 0.036 | 0.79 (0.55 – 1.14) | 0.201 |
| ≥2 vs. 0 | 1.82 (  1.08 – 3.06  ) | 0.025 | 1.71 (1 – 2.92) | 0.049 | 1.72 (1.1 – 2.68) | 0.016 | 1.77 (1.13 – 2.78) | 0.011 |
| Smoking (ever) | 0.83 (  0.61 – 1.12  ) | 0.229 |  |  | 1.13 (0.88 – 1.46) | 0.341 |  |  |
| FEV1% | 0.99 (  0.98 – 1  ) | 0.034 | 0.99 (0.98 – 1) | 0.222 | 1 (0.99 – 1) | 0.372 |  |  |
| DLCO% | 0.99 (  0.98 – 1  ) | 0.034 | 1 (0.99 – 1.01) | 0.934 | 1 (0.99 – 1) | 0.268 |  |  |
| Neoadjuvant therapy | 1.27 (  0.87 – 1.85  ) | 0.209 |  |  | 1.3 (0.94 – 1.8) | 0.107 |  |  |
| Histology (squamous) | 1.19 (  0.84 – 1.69  ) | 0.319 |  |  | 1.31 (0.98 – 1.75) | 0.066 |  |  |
| Location |  |  |  |  |  |  |  |  |
| RML vs. RUL | 1.63 (  0.4 – 6.64  ) | 0.496 |  |  | 1.3 (0.32 – 5.27) | 0.712 |  |  |
| RLL vs. RUL | 1.31 (  0.73 – 2.37  ) | 0.366 |  |  | 1.47 (0.91 – 2.36) | 0.116 |  |  |
| LUL vs. RUL | 1.17 (  0.82 – 1.68  ) | 0.396 |  |  | 1.14 (0.84 – 1.55) | 0.395 |  |  |
| LLL vs. RUL | 1.29 (  0.86 – 1.93  ) | 0.222 |  |  | 1.17 (0.82 – 1.67) | 0.382 |  |  |
| Angioplasty | 1.36 (  0.83 – 2.25  ) | 0.225 |  |  | 1.52 (0.99 – 2.33) | 0.057 |  |  |
| R1 | 1.07 (  0.7 – 1.62  ) | 0.763 |  |  | 1.24 (0.89 – 1.73) | 0.205 |  |  |
| LN number | 0.98 (  0.96 – 1.01  ) | 0.141 |  |  | 0.99 (0.97 – 1.01) | 0.473 |  |  |
| Pathological T stage |  |  |  |  |  |  |  |  |
| T3 vs. T2 | 1.26 (  0.81 – 1.96  ) | 0.303 | 1.37 (0.87 – 2.15) | 0.173 | 1.07 (0.71 – 1.6) | 0.747 | 1.27 (0.92 – 1.76) | 0.140 |
| T4 vs. T2 | 2.44 (  1.28 – 4.64  ) | 0.007 | 2.17 (1.13 – 4.18) | 0.020 | 2.35 (1.34 – 4.13) | 0.003 | 2.58 (1.88 – 3.53) | < 0.001 |
| Pathological N stage |  |  |  |  |  |  |  |  |
| N1 vs. N0 | 1.24 (  0.87 – 1.78  ) | 0.242 | 1.33 (0.91 – 1.92) | 0.138 | 1.32 (0.97 – 1.8) | 0.081 | 1.27 (0.92 – 1.76) | 0.143 |
| N2 vs. N0 | 2.02 (  1.41 – 2.91  ) | < 0.001 | 2.21 (1.52 – 3.23) | < 0.001 | 2.7 (1.99 – 3.66) | < 0.001 | 2.58 (1.88 – 3.53) | < 0.001 |
| Adjuvant therapy | 0.71 (  0.53 – 0.96  ) | 0.027 | 0.69 (0.51 – 0.95) | 0.023 | 1.43 (1.11 – 1.84) | 0.006 | 1.35 (1.04 – 1.75) | 0.031 |

OS, overall survival; PFS, progression-free survival; HR, hazard ratio; CI, confidence interval; BMI, body mass index; FEV1, forced expiratory volume in 1 second; DLCO, carbon monoxide diffusing capacity; CCI, Charlson Comorbidity Index; RUL, right upper lobe; RML, right middle lobe; RLL, right lower lobe; LUL, left upper lobe; LLL, left lower lobe.
